# Formulation-Dependent Extrudability of Highly Filled Alginate System for Vaginal Drug Delivery

**DOI:** 10.3390/gels11070510

**Published:** 2025-07-01

**Authors:** Arianna Chiappa, Alice Fusari, Marco Uboldi, Fabiana Cavarzan, Paola Petrini, Lucia Zema, Alice Melocchi, Francesco Briatico Vangosa

**Affiliations:** 1 Dipartimento di Chimica, Materiali e Ingegneria Chimica “G. Natta”, Politecnico di Milano, Piazza Leonardo da Vinci 32, 20133 Milano, Italy; arianna.chiappa@polimi.it (A.C.); alice.fusari@polimi.it (A.F.); fabiana.cavarzan@polimi.it (F.C.); paola.petrini@polimi.it (P.P.); 2 PhormulaMi Lab, Sezione di Tecnologia e Legislazione Farmaceutiche “Maria Edvige Sangalli”, Dipartimento di Scienze Farmaceutiche, Università degli Studi di Milano, Via Giuseppe Colombo 71, 20133 Milano, Italy; marco.uboldi@unimi.it (M.U.); lucia.zema@unimi.it (L.Z.); alice.melocchi@unimi.it (A.M.)

**Keywords:** alginate-based hydrogel, high solid powder content hydrogel, rheological characterization, drug delivery

## Abstract

The incorporation of solid particles as a filler to a hydrogel is a strategy to modulate its properties for specific applications, or even to introduce new functionalities to the hydrogel itself. The efficacy of such a modification depends on the filler content and its interaction with the hydrogel matrix. In drug delivery applications, solid particles can be added to hydrogels to improve drug loading capacity, enable the inclusion of poorly soluble drugs, and modulate release kinetics. This work focuses on the case of alginate (ALG)-based hydrogels, obtained following an internal gelation procedure using CaCO_3_ as the Ca^2+^ source and containing a high solid volume fraction (up to 50%) of metronidazole (MTZ), a drug with low water solubility, as a potential extrusion-based drug delivery system. The impact of the hydrogel precursor composition (ALG and MTZ content) on the rheological behavior of the filled hydrogel and precursor suspension were investigated, as well as the hydrogel stability and MTZ dissolution. In the absence of solid MTZ, the precursor solutions showed a slightly shear thinning behavior, more accentuated with the increase in ALG concentration. The addition of drugs exceeding the saturation concentration in the precursor suspension resulted in a substantial increase (about one order of magnitude) in the low-shear viscosity and, for the highest MTZ loadings, a yield stress. Despite the significant changes, precursor formulations retained their extrudability, as confirmed by both numerical estimates and experimental validation. MTZ particles did not affect the crosslinking of the precursors to form the hydrogel, but they did control its viscoelastic behavior. In unfilled hydrogels, the ALG concentration controls stability (from 70 h for the lowest concentration to 650 h for the highest) upon immersion in acetate buffer at pH 4.5, determining the MTZ release/hydrogel dissolution behavior. The correlations between composition and material properties offer a basis for building predictive models for fine-tuning their composition of highly filled hydrogel systems.

## 1. Introduction

Hydrogels loaded with a high amount (>20%) of suspended micro- or nanoparticles are also referred to as suspension-based, highly loaded, or particulate-laden hydrogels, and they fall within the category of highly filled materials [1].

Therapeutic agents can be used as fillers in pharmaceutical hydrogel formulations [2]. These systems belong to drug delivery systems (DDSs) and are increasingly emerging as one of the most promising and technologically advanced therapeutic approaches across various clinical scenarios, as they enhance treatment efficacy, including cancer therapies and [3] wound healing [4].

The fillers, especially if in high concentration, modulate the mechanical properties of hydrogels, which enhances local retention, sustains a site-specific drug release [5], and minimizes drug off-target accumulation throughout the body, thus reducing adverse effects associated with systemic exposure [6,7]. The release of drugs can be modulated through multiple mechanisms, including diffusion and swelling, as well as in response to external stimuli such as pH changes, temperature, and the presence of enzymes [8].

Highly filled hydrogels overcome the water solubility issues that are so common for many drugs, making it possible to load poorly water-soluble drugs at a therapeutic dosage in a water-based system: the hydrogel. They are particularly valuable in mucosal, dermal, and implantable drug delivery, where prolonged drug availability and high local concentrations are desirable, and solubility limits often necessitate formulations containing drugs above their saturation concentration [9].

In this context, the use of extrudable hydrogels, soft materials that can pass through a nozzle, are gaining increasing attention for the 3D printing of dosage forms in personalized medicine, and minimally invasive administration, where control of flow is essential [10]. Despite their potential, the formulation of such systems remains challenging: the presence of solid particles significantly influences the mechanical properties of the hydrogel [11] in such a way that upon extrusion through a nozzle the hydrogel may undergo damage.

A strategy to promote highly filled drug delivery systems (HF-DDS) extrudability is by injecting the liquid hydrogel precursors, which are converted in situ into a hydrogel via safe physical or chemical crosslinking [12,13].

To date, a variety of in situ-forming injectable hydrogels have been developed for gene delivery, regenerative medicine, and cell therapy [14,15,16]. Among the hydrogel-forming polymers that can be exploited for in situ crosslinking, alginate (ALG) represents one of the most investigated [17,18,19,20] due to the compatibility of the crosslinking process to the in situ delivery.

A specific mechanism of crosslinking, referred to as internal gelation, can be controlled so that the alginate crosslinking kinetic can be adjusted to be extruded, either by injection or 3D printing, in a viscous liquid form, while progressing to the formation of a shape-defined gel after extrusion [21,22]. One of the challenges of extrusion is selecting an appropriate process timing for the extrusion hydrogel precursor in its liquid phase. Even in this case, however, the addition of a high volumetric fraction of micro- or nanoparticles to the precursor solution increases its viscosity, thus limiting or even suppressing precursor extrudability. This may require increasing the nozzle size and/or the applied pressure to extrude the hydrogel precursor.

Besides the extrudability issue, common to several application fields and technologies, for the specific case of Drug Delivery Systems (DDS), the stability of the hydrogel and the release mechanism and kinetics are of the utmost importance, and this aspect may also be affected by the hydrogel composition and particle volume fraction. 

In this work, we propose a systematic investigation of how hydrogel composition and microparticle loading affect hydrogel precursor rheology and filled hydrogel mechanical properties and stability, with the aim of providing practical tools for optimizing both formulation and device efficiency.

As a case study, the investigation is carried out on an alginate (ALG) hydrogel obtained via internal gelation and filled with metronidazole (MTZ), in view of its use as DDS for the first-line treatment of bacterial vaginosis in the vaginal environment. Indeed, this environment poses a great challenge for drug delivery systems, due to the low pH and the requirements of sustained release. In respect to the first challenge, thanks to its good stability in a pH range from 3 to 10, ALG represents an interesting candidate for the development of hydrogel-based drug delivery systems intended for vaginal delivery [23]. However, the resulting systems could have some drawbacks due to their softness/compliance, and their limited adhesion to the mucosa and therefore relatively short residence time, especially considering that the continuous renewal of fluids might contribute to the removal of the dosage form [24,25]. Furthermore, when MTZ is considered as an active ingredient, its low aqueous solubility of MTZ (12.476 mg/mL) makes it difficult to achieve sustained therapeutic levels. The adoption of a filled hydrogel as DDS would on one hand permit the appropriate dosage of MTZ, and on the other hand would allow modulation of the DDS mechanical and release performance. Finally, control of hydrogel precursor solution rheological behavior would allow its adoption in different dosage scenarios, from dispensing to the preparation of cast or 3D-printed soft drug delivery devices.

## 2. Results and Discussion

### 2.1. Metronidazole Particle Size Distribution

**Figure 1:**
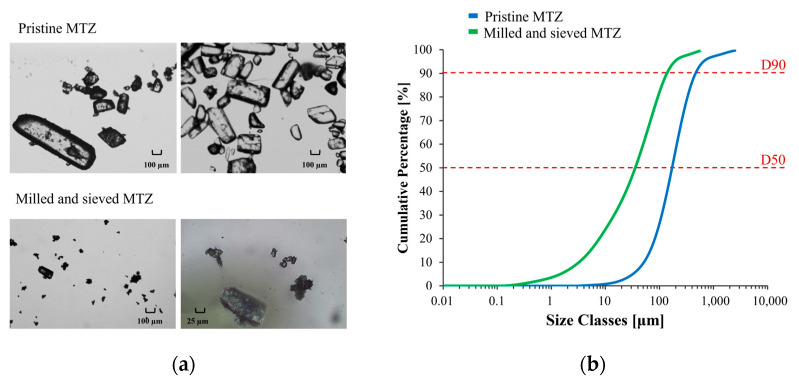
Optical micrographs (**a**) and cumulative particle size distribution curves of pristine and milled/sieved MTZ (**b**).

The granulometric profile of pristine MTZ was determined to evaluate the particle size distribution of the powder and to rule out the larger particles, which could also be observed by optical microscopy (Figure 1a). Considering that the apparent D90 value of pristine MTZ was equal to 0.497 mm, the risk of clogging phenomena during any extrusion operation, either performed via syringe or using the 3D printer, could not be excluded. For this reason, the material was milled and further sieved. This way, the apparent D90 was reduced to 0.146 mm, thus limiting the risk of clogging (Figure 1b).

### 2.2. Solutions and CaCO_3_ Suspensions Flow Behavior Without MTZ Powder

**Figure 2:**
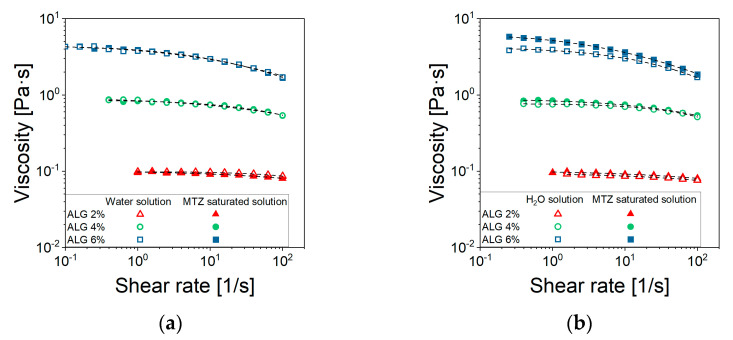
Viscosity curves of (**a**) ALG solutions and (**b**) CaCO_3_-suspensions prepared using either water (□) or a saturated solution of MTZ (■). Each data point represents the average of at least 5 tests. For the sake of readability, standard deviations are omitted, being very limited. The excellent data reproducibility can be observed in the Supplementary Information, Figure S1.

In steady state shear tests of ALG solutions, prepared either using distilled water or a saturate MTZ solution (Figure 2a), raising the ALG concentration resulted in an increase in the zero-shear viscosity value, $\eta_{0}$, and lowered the shear rate level at which the shear thinning behavior can be observed. This latter effect was especially noted for solutions containing 4% and 6% ALG. Instead, a quasi-Newtonian behavior was observed for 2% ALG solution.

**Figure 3:**
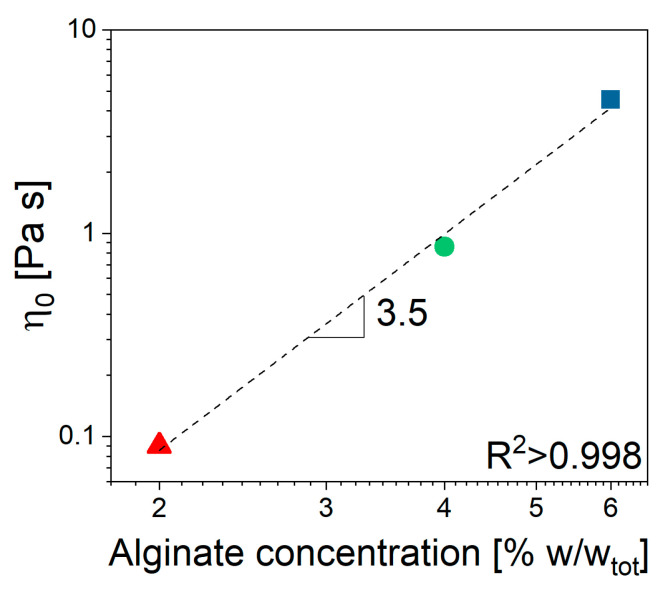
Unfilled alginate solution. Zero shear viscosity dependence on ALG concentration.

Furthermore, there was no significant interaction between ALG and the selected drug, despite the ionic nature of both species (Figure 2a), as the viscosity seemed not to be affected by the presence of MTZ in solution. All viscosity data could be fitted using the Cross’ model (Equation (5)) with the parameter reported in Table 1. Despite the 2% ALG solution, the determination of the *m* and *l* parameters is quite uncertain, given its quasi-Newtonian behavior. The most significant effect of ALG concentration, *C_ALG_*, consisted of an increase in $\eta_{0}$ (Figure 3) according to Equation (1):

(1) $$\eta_{0}=A\times {C_{ALG}}^{n}$$

The value 3.5 of the power low index, *n*, indicated that in the given range of concentrations, the solution was in the entangled regime, according to Takahashi [26]. When CaCO_3_ is dispersed in the solution containing 6% ALG (NA_C.0), a slight increase in viscosity at fixed shear rate can be observed, while no effect can be detected for solutions containing 2% and 4% ALG (Figure 2b).

### 2.3. MTZ Suspensions Flow Behavior

**Figure 4:**
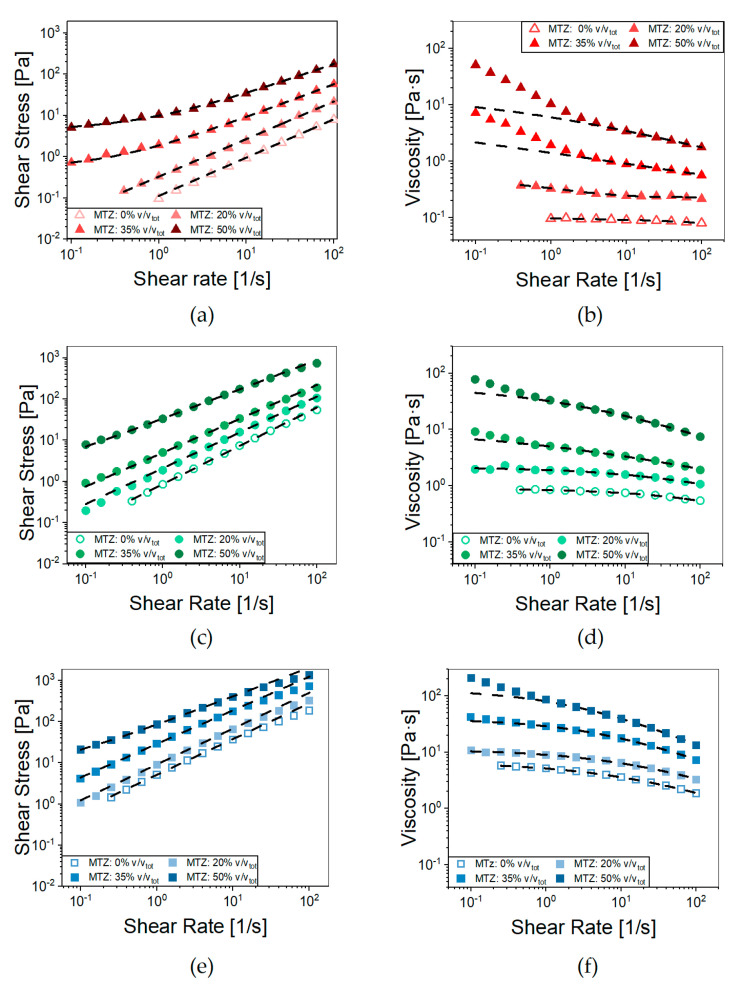
Flow curves of (**a**) NA_A, (**c**) NA_B, and (**e**) NA_C containing increasing volumetric fractions of MTZ powder (0–50%). Dashed lines represent the fitting with the Herschel–Bulkley model (Equation (6)). Viscosity curves of (**b**) NA_A, (**d**) NA_B, and (**f**) NA_C containing increasing amounts of MTZ powder (0–50%); dashed lines represent their fitting with the Cross’ Model (Equation (5)). Each data point represents the average of at least 5 tests. For the sake of readability, standard deviations are omitted.

The effects of adding MTZ powder above the limit of its solubility were manifold, and their impact depended also on the ALG concentration (Figure 4).

For all the ALG concentrations, when increasing the MTZ content, there was an upward shift in the viscosity curves at medium-to-high shear rate. In this range, the Cross’ model fits the data with the parameters reported in Table 2. A second effect was the rise in the viscosity with decreasing shear rates in the presence of high MTZ volume fractions. Indeed, the substructures, due to particle interactions present in quiescent conditions, required the application of a shear stress above a certain threshold to be disaggregated, so that flow can occur. This effect was present in all formulations but was particularly evident for the A suspensions (NA_A.X—Section 4.2.2 Hydrogel preparation introduces the code adopted to designate the materials) where the Herschel–Bulkley model (Equation (6)) accurately fitted the stress–shear rate curves (R^2^ > 0.999) over the entire shear rate range considered (Figure 4b, Table 3).

Besides increasing the suspension consistency coefficient, (*k*), and the yield stress threshold value, *τ_y_*, raising the content of MTZ solid particles led to a more pronounced shear thinning, as suggested by the viscosity index (*n*) trend. This behavior can be due to particle orientation in the flow direction under intense shear, which is typical of suspensions characterized by limited interactions between the solid particles and the liquid phase [27]. A further mechanism favoring shear thinning is the breakdown of agglomerates; however, in this specific case, no clear sign of agglomeration was observed for the samples (see Figure S2). In any case, the contribution to shear thinning of some (hindered) interaction between the particles at high filler volume fraction cannot be excluded. From the extrudability standpoint, the presence of a yield stress threshold may imply exceedingly high extrusion pressures, especially in the case of long or thin nozzles/needles, as discussed in the following. On the other hand, it may be useful when the extrusion process is performed in the direction of gravitational forces, such as in 3D printing, because it prevents dripping.

**Figure 5:**
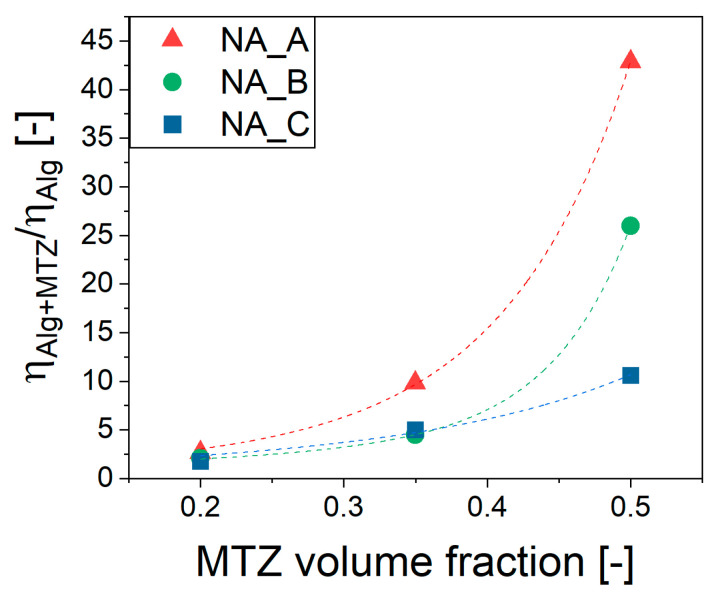
Viscosity dependence on MTZ volume fraction of ALG suspensions. Dashed lines are the Thomas’ equation (Equation (2)) fitting. The viscosity data considered is that measured at 10 s^−1^.

The effect of MTZ volume fraction, ϕ, on the viscosity of NA_suspensions was apparent from Figure 5, in which an increase of over 40 times the viscosity of unfilled precursor solution was observed for the 2% ALG. The effect was lower, but still very important for the 4% ALG and 6% ALG solution.

This highly non-linear effect was well captured by the Thomas equation (Equation (2)) [28]:

(2) $$\eta_{NA_{A}.X}\left(\dot{\gamma }=10s^{.1}\right)=\eta_{NA_{A}.0}\left(\dot{\gamma }=10s^{.1}\right)\times \left(1\;+\;2.5\;\phi \;+\;10.05\phi^{2\;\;}+\;A^{B\phi }\right)$$

where *ϕ* is the MTZ volume fraction, and *A* and *B* are empirical coefficients (Table 4).

### 2.4. Extrudability

**Figure 6:**
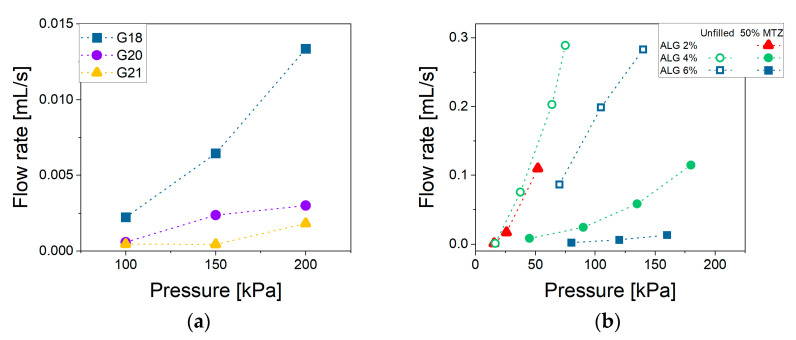
Flow rate vs. pressure for (**a**) NA_C.50 extruded through G18, G20, G21 nozzles and (**b**) NA_suspensions containing different MTZ amounts extruded through the G18 nozzle. Lines are intended as a visual aid. Data for the suspension containing ALG 2% are unavailable, as its low viscosity caused uncontrolled dripping from the cartridge even under atmospheric pressure.

The material ability to flow through a small opening under the application of pressure is defined here as extrudability. This parameter is strictly related to the viscosity which, in the case of non-Newtonian fluids, depends on the shear rate applied. Generally, in an extrusion process, the shear rates could range between 100 and 10,000 s^−1^, which is definitely higher than the typical range explored through rotational rheometer experiments [29]. To properly estimate the viscosity in the above-mentioned range, it is possible to extrapolate a suitable rheological model to the shear rates of interest. This procedure may, however, be a source of errors. A more appropriate approach would be to experimentally extend the measurement range, adopting other rheological techniques. In this work, we propose the use of a 3D bioprinter as a capillary rheometer, following the procedure described in Section 4.2.4. By way of example, Figure 6a reports the flow rate’s dependence on extrusion pressure and nozzle diameter for the most filled NA_suspension (NA_C.50). Figure 6b instead highlights the effect of the MTZ content at fixed nozzle diameter. It can be noted that at all imposed pressures the flow rate was higher for the NA_suspensions with lower viscosity. When considering NA_X.50 suspensions, a deviation from linearity could be observed due to the shear thinning behavior.

**Figure 7:**
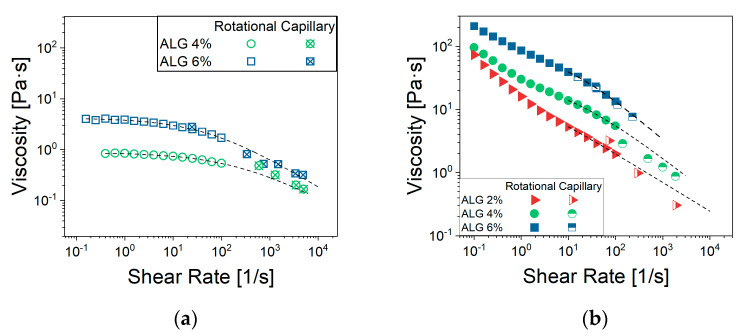
Flow curves for NA_suspensions (**a**) without MTZ-powder and (**b**) containing 50% of MTZ. For suspensions with 2% ALG, data could not be acquired due to spontaneous outflow from the cartridge.

Data of Figure 6b allowed us to estimate the viscosity at shear rates typical of the extrusion process by applying the method described in Section 4.2.4. For NA_C formulation, the data from rotational and capillary rheometry measured at the same shear rate overlap (Figure 7). The overlap confirmed the validity of the adopted method, including the procedures and assumptions adopted in data elaboration as reported in Section 4.2.4, at least for the case of NA_C suspensions. As for NA_A and NA_B, direct comparison between data at the same shear rate was not possible, and the agreement of experimental data and Cross’ rheological model (Equation (5)) with parameters from Table 2 predictions is limited, even if still acceptable.

Based on the proposed approach, once the rheological behavior of a suspension is known, its extrudability can be predicted, at least to an acceptable approximation, by the comparison of the estimated pressure/force required to extrude the material though a nozzle of any given diameter with the maximum pressure or force available for the extrusion process—as an example, about 80 N in the case of manual extrusion from a syringe. Furthermore, in case of automated extrusion processes, such as 3D bioprinting, the rational approach proposed allows us to select the appropriate pressure for the desired extrusion rate with a limited need to resort to time-consuming and expensive trial-and-error sessions. Obviously, the approach validity is limited to the cases in which no agglomeration or nozzle clogging occurs. The second effect may be critical for clinical nozzles (<0.3 mm diameter) and suspension filled with particles with comparable dimensions.

### 2.5. Dynamic Mechanical Response ALG Based Hydrogels

**Figure 8:**
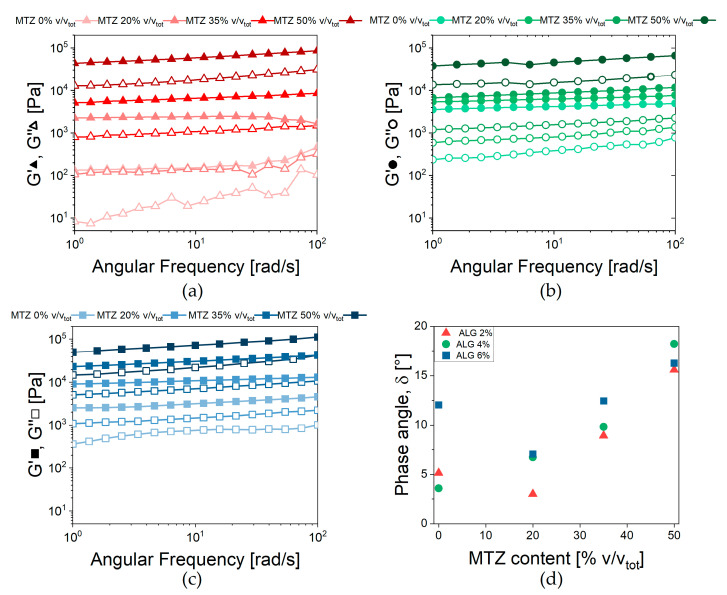
Viscoelastic behavior of ALG-based hydrogels. Storage (G’) and loss (G”) moduli vs. frequency for hydrogels containing (**a**) 2% Alginate, (**b**) 4% Alginate, and (**c**) 6% Alginate. (**d**) Phase shift angle measured at 2.5 rad/s for all the formulations tested.

The results of frequency sweep tests in Figure 8 confirm that, after GDL addition, a hydrogel was formed for any MTZ content, as confirmed by the limited dependence on frequency and the low level of the phase shift angle. Also, the crosslinking kinetics were practically unaffected by the filler: the gel point was reached after about 30 min from adding GDL, while the reaction ended after about 4 h (Figure S3).

Focusing on unfilled hydrogel, G’ and G” increased with increasing ALG concentration, due to the increase in the Ca^2+^ links between alginate chains. To capture this effect, the average mesh size *ξ* [30,31] was obtained from Equation (3):

(3) $$\zeta \;=\;{\left(\frac{G_{0}\cdot N_{A}}{RT}\right)}^{-\frac{1}{3}}$$

where $G_{0}$, the fully relaxed hydrogel modulus, was approximated to G’, given the quasi-elastic behavior of the hydrogel, *N_A_* is the Avogadro constant, *R* is the gas constant, and *T* is the absolute temperature, in this case 298.15 K. The parameter, inversely proportional to the distance between two crosslinks, is equal to 290 nm, 98 nm, and 110 nm for HA, HB, and HC unfilled hydrogels, respectively.

The same approach was not applied to the filled hydrogels, as the MTZ are not believed to directly affect the degree of crosslinking. Rather, based on the transient nature of the ionic crosslinks, the limited dependence of the viscoelastic behavior on angular frequency, and the low phase angle, the Weak Gel model [32] (Equation (4)) was adopted:

(4) $$G^{*}\left(\omega \right)=A_{f}\omega^{\frac{1}{z}}$$

This model describes the gel as constituted by flowing units—in the present case, the alginate polymeric chains—interacting with each other and the other components of the complex system. The parameter *A_f_* is related to the strength of interaction between flowing units, and the scaling exponent *z* gives an indication of the number of interactions between them.

**Figure 9:**
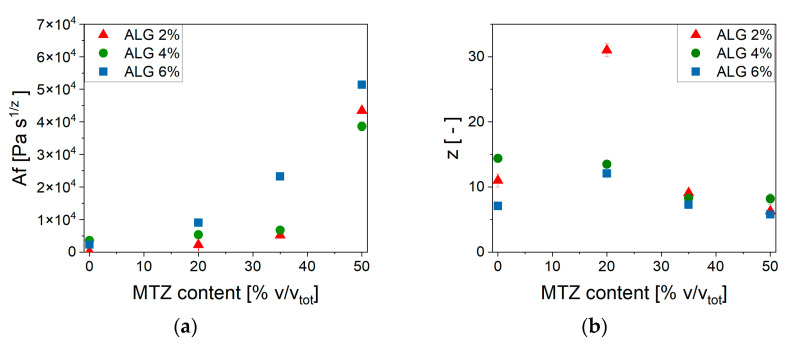
Weak gel model parameters’ dependence on MTZ volume fraction. (**a**) *Af*; (**b**) *z*.

The parameters’ dependence on MTZ concentration can be observed in Figure 9.

At each ALG concentration, the values of *A_f_* increased with increasing MTZ content and reached a comparable value for all ALG concentration. On the other hand, the number of interactions between alginate chains, *z*, was, in general, reduced at increasing MTZ volumetric fractions. In general, MTZ appeared to dominate the rheological behavior of the hydrogel by stiffening it, probably due to the inherent stiffness of the MTZ particle, but also modifying the interactions between the macromolecules forming the hydrogel. This is also associated with an increase in the dissipative behavior of the filled hydrogel, as shown by the increase in the phase angle, *δ* (Figure 8d), whose cause is still to be identified.

### 2.6. Swelling Behavior and Stability

**Figure 10:**
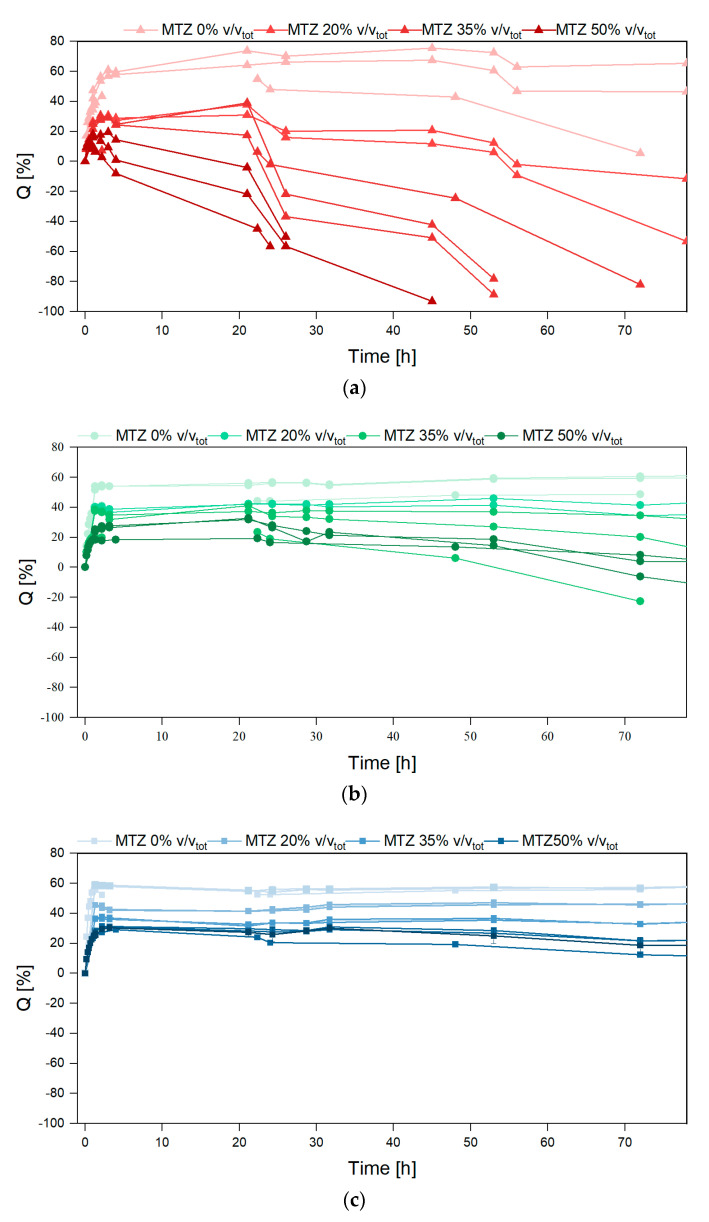
Swelling ratio vs. time for hydrogels containing (**a**) ALG 2%, (**b**) ALG 4%, and (**c**) ALG 6%.

**Figure 11:**
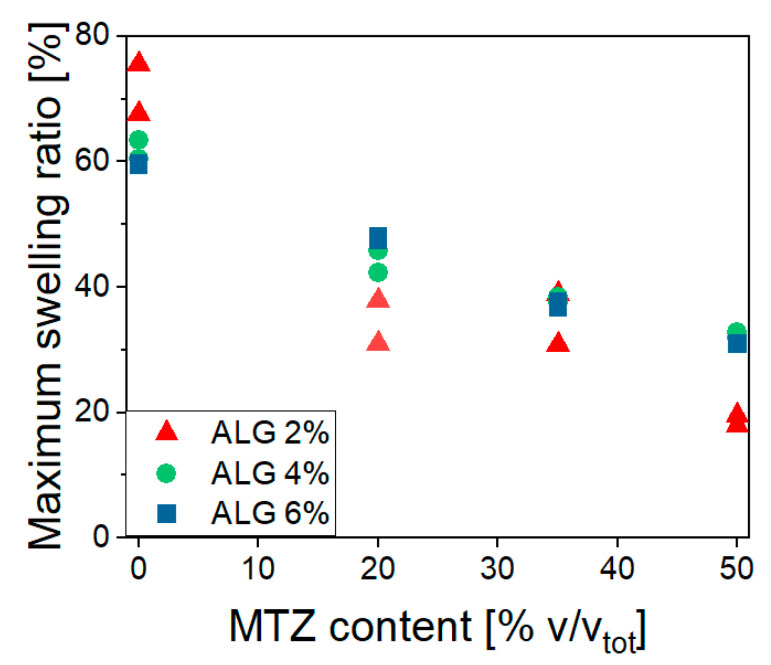
Maximum swelling ratio dependence on ALG and MTZ content.

Figure 10 shows a fast buffer solution uptake occurring in the first hour, causing an increase in the mass of all the investigated hydrogels. This uptake magnitude is highlighted in Figure 11, showing the effects of ALG and MTZ contents. In unfilled hydrogels, the uptake is consistent with the mesh measurement, as expected. At each ALG concentration, the swelling ability decreased with the MTZ fraction, as the hydrogel volume might be gradually occupied by drug particles, which hindered the acetate buffer entrance.

**Figure 12:**
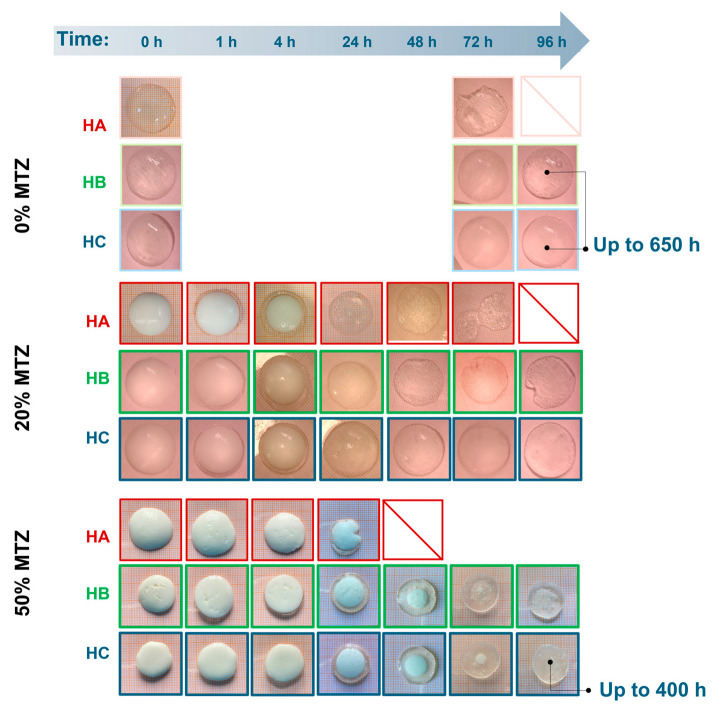
Images of samples extracted from the buffer solution at different time points of immersions.

After the maximum swelling ratios, *Q* was reached, and different behavior was observed based on the amount of MTZ powder. Unfilled hydrogels (HB.0 and HC.0) showed a limited deswelling followed by a plateau, corresponding to an equilibrium state for the whole experiment duration (650 h). HA.0 showed a significant reduction in the swelling ratio, which is not related to a loss of buffer solution mass, but to the disaggregation of the hydrogel sample, as also evident from images in Figure 12. This phenomenon is probably due to the presence of sodium ions in acetate buffer, which competed with calcium ions and led to a reduction in the crosslinking degree, followed by dissolution on the un-crosslinked alginate chains [33]. With increasing solid fraction content, all the hydrogels showed a reduction in the swelling ratio, to be interpreted in terms of ALG/MTZ mass loss. This phenomenon was faster for hydrogels containing less ALG (at a fixed MTZ content), or more MTZ (at a fixed ALG concentration). On one hand, the lower stability of the ALG hydrogel may cause a combined MTZ release and filled gel disaggregation, but on the other, the solubilization of MTZ powder may generate cavities that weaken the structure and foster sample fragmentation.

This is made clearer from images of samples extracted from the buffer solution at different time points (Figure 12). The clearness of the hydrogel matrix allowed the direct observation of the MTZ dissolution, with the front moving towards the center of the sample. Indeed, HA.50 hydrogel MTZ dissolution also involved hydrogel fragmentation, whereas HB.50 and HC.50 remained intact while dissolution occurred, suggesting drug solubilization and diffusion through a swollen hydrogel layer mechanism. Comparing the last two, the presence of a small portion of MTZ powder in HC.50 at 72 h might indicate a slower dissolution when a higher alginate content was present, which is consistent with the denser polymeric network for HC hydrogels. However, further investigation evidenced no differences in the dissolution profile of these hydrogels and demonstrated that the dissolution was governed by the solubility of the active ingredient which belongs to the BCS II class [34]. In all cases, for the investigated geometry, the MTZ dissolved completely after about 4 days of immersion in the buffer solutions. These results reflect the behavior of the hydrogels at pH 4.5, a reference value commonly [35] used in the context of bacterial vaginosis. However, since vaginal pH may vary between individuals, future studies should consider the impact of pH on drug release in the perspective of personalized therapy.

## 3. Conclusions

This study demonstrates that the addition of solid fillers impacts on the rheological behavior of hydrogels and their liquid precursors, on the hydrogel stability, and on the release of the filler, with implications for the design of extrusion-based drug delivery systems. Rather than developing a specific drug delivery system, we used a representative case to prove how filler incorporation affects hydrogel behavior, aiming to inform broader strategies for modeling and designing filled hydrogel systems.

This was explored through the specific case of alginate hydrogels filled with metronidazole (MTZ), a possible sustained release system for the first-line treatment for bacterial vaginosis. The addition of MTZ, up to a volumetric fraction of 0.5, significantly altered the flow behavior of the precursors of hydrogels. The effect is not only limited to a more-than-linear increase in shear viscosity at low shear rates but also involves a shift in the behavior of the complex system from (non-linear) viscous to Bingham-like, with a threshold shear stress required for flow. These changes have clear implications for both extrudability and shaping processes like extrusion or 3D printing.

Notably, extrudability is not determined only by hydrogel formulation, but also by boundary conditions such as nozzle geometry and applied pressure/driving force. A simple method was proposed to estimate the required extrusion pressure using basic fluid dynamics, with improved reliability when measurements reflect actual processing conditions.

Despite MTZ incorporation, the precursor suspension is extrudable even at the higher MTZ content. and the crosslinking reaction was not prevented. Through filler addition, the hydrogel properties can be tuned from those of a quite soft material to those of a stiff, self-standing one, indicating the possibility to modulate gel structure through filler addition.

While the findings are specific to the alginate–metronidazole system in a buffered solution, this work underscores the importance of systematically linking material formulation and flow behavior, setting up a framework for designing filled hydrogel-based delivery systems. This approach is particularly relevant in the context of personalized medicine, where no single formulation is likely to suit all clinical scenarios of bacterial vaginosis. Instead, different therapeutic needs may require tailored compositions with distinct rheological and release characteristics. Our study therefore provides formulation insights and practical tools to support the selection and design of systems best suited to individual requirements.

## 4. Materials and Methods

### 4.1. Materials

Sodium alginate (ALG; average Mw = 120,000–190,000 g/mol, M/G ratio = 1.56, Merk KGaA, Darmstadt, Germany); calcium carbonate, in the form of calcite (CaCO_3_; Merk KGaA, Darmstadt, Germany); glucono-δ-lactone (GDL; Merk KGaA, Darmstadt, Germany); metronidazole (MTZ; Mw = 171.15 g/mol, density = 1.45 g/mL, Methapharmaceutical, Barcelona, Spain).

### 4.2. Methods

#### 4.2.1. Metronidazole Preparation

MTZ particle size was reduced using a pin mill (Retsch ZM200, Retsch GmbH, Haan, Germany) equipped with a 250 μm mesh filtering-screen and by setting the rotation speed at 6000 rpm for 5 s.

The <180 μm fraction was selected. The particle size distribution of MTZ was assessed (Mastersizer 3000, Malvern Panalytical, Worcestershire, UK) before and after the milling process. Images of pristine and processed MTZ were acquired using an optical microscope (5× and 20× magnification, Olympus BX 60, Olympus Global, Tokyo, Japan).

#### 4.2.2. Hydrogel Preparation

The hydrogel was attained by using a previously set-up protocol entailing an internal gelation process [34,36]. The developed procedure required addition of previously weighted (E50S/3, Gibertini Elettronica, Milano, Italy) amounts of CaCO_3_ and ALG in a saturated solution of MTZ in distilled water with the formation, under magnetic stirring (300 rpm for about 30 min at room temperature), of a suspension, here referred to as CaCO_3_-suspensions. When needed, MTZ powder was added in different volume fractions (20, 35, 50% V/V_tot_) to the CaCO_3_ suspension and manually mixed for 2 min, producing the drug-loaded suspension. During preparation the mechanical stirring was carried out so as to avoid particle aggregation phenomena. Figure S2 shows a micrograph of the particles incorporated in the hydrogel, whose dimensions are comparable to those of dry particles after milling and sieving. Ca^2+^ ions were made slowly available by adding the selected acidifying agent (i.e., GDL 3.6% w/w_tot_) dissolved in an MTZ-saturated solution to the drug-loaded suspension, resulting in the formation of a GDL-activated suspension. The solution containing the acidifying agent was added in a 1:2 volume ratio with respect to the previously prepared CaCO_3_-suspension, thus diluting its ingredients. This way, the hydrogel precursor formulation remained in the liquid state for approximately 30 min, while the complete crosslinking of the hydrogel required about 4 h.

When still extrudable (not less than 1 h and not more than 2 h from preparation), the hydrogel precursor formulations were extruded using either a 5 mL syringe equipped with G18 gauge (internal diameter = 0.84 mm) needles, attaining dome-shape samples, or an extrusion-based bioprinter (BIO X, CELLINK, Gothenburg, Sweden) equipped with a 3 mL cartridge and with G18 gauges to obtain 3D printed samples.

The final composition of all the hydrogels evaluated in this work, differing for the content of ALG, CaCO_3_, and MTZ, is summarized in Table 5. Instead of testing the GDL-activated suspensions, which have time-dependent properties, not-activated suspensions (indicated with the prefix tag “NA_”) having the same composition except for the presence of GDL were considered when needed.

#### 4.2.3. Rotational Rheological Measurement on Solutions and Suspensions

Shear viscosity curves were measured using a Modular Compact Rheometer MCR 502 (Anton Paar, Graz, Austria) in a parallel plate configuration (25 mm or 50 mm diameter plates, 1.5 mm gap). Steady shear test measurements were performed by imposing a shear rate logarithmic ramp from 0.1 s^−1^ to 100 s^−1^ for each formulation. The test temperature was 25 °C.

The shear-thinning behavior of tested samples was fitted by Cross’ model (Equation (5)).

(5) $$\tau (\dot{\gamma })=\eta (\dot{\gamma })\times \dot{\gamma }\;\frac{\eta (\dot{\gamma })-\eta_{\infty }}{\eta_{0}-\eta_{\infty }}=\frac{1}{{1+(\lambda \dot{\gamma })}^{m}}$$

where *τ* is the shear stress, $\eta (\dot{\gamma })$ is the shear rate dependent viscosity, $\dot{\gamma }$ the shear rate, $\eta_{0}$ and $\eta_{\infty }$ are the viscosities at zero and infinite shear rate, respectively, *λ* is characteristic relaxation time, and m is the power law index.

In this work, $\eta_{\infty }$ was assumed to be equal to zero. For some suspensions, yielding phenomena occurred and the relevant shear stress and shear rate curves were fitted by the Herschel–Bulkley model (Equation (6)).

(6) $$\tau (\dot{\gamma })=\tau_{y\;}+{k\dot{\gamma }}^{n}$$

where $\tau_{y\;}$is the yield stress, *k* the fluid consistency, and *n* the viscosity index.

#### 4.2.4. Extrudability/3D Printability

The possibility of extruding/3D printing the MTZ-filled hydrogel precursor formulations was evaluated with an extrusion-based bioprinter (BIO X, CELLINK, Gothenburg, Sweden) equipped with a 3 mL cartridge. This device is a pressure-controlled 3D printer and could be used to assess the minimum pressure required to extrude the considered NA_suspensions through 19 mm long G18, G20, and G21 needles, having an internal diameter of 0.84 mm, 0.61 mm, and 0.51 mm, respectively.

Furthermore, the device was also used as a capillary rheometer to estimate the rheological behavior of the hydrogel precursor formulations at extrusion-relevant shear rates [37]

The apparent shear stress at the wall *τ_w_* was related to the pressure drop through the bioprinter needle, $P_{c}$:

(7) $$\tau_{w}=\frac{R_{c}P_{c}}{2L_{c}}$$

where $R_{c}$ and $L_{c}$ are, respectively, the radius and the length of the needle.

The pressure drop in the capillary was calculated from the pressure imposed on the bioprinter piston, $P_{b}$, as:

(8) $$P_{c}=P_{b}-P_{e}$$

where $P_{e}$ is the pressure drop due to converging flow at the needle entrance, estimated in independent extrusion experiments performed without needles. $P_{e}$ was assumed to be shear rate independent. The pressure drops along the reservoir/cartridge was neglected, given a ratio between nozzle and reservoir diameter of order 3.

Assuming the parabolic velocity field typical of Newtonian fluids in a cylindrical channel, the shear rate at wall, ${\dot{\gamma }}_{w,Newt}$, is related to the flow rate *Q* by Equation (9).

(9) $${\dot{\gamma }}_{w,Newt}=\frac{4Q}{\pi R_{c}^{3}}$$

For non-Newtonian fluids, this value represents the apparent shear rate, a lower estimation of the true value. An apparent shear viscosity *η_app_* can be then calculated (Equation (10)):

(10) $$\eta_{app}=\frac{\tau_{w}}{{\dot{\gamma }}_{w,Newt}}$$

In the case of Newtonian fluids, $\eta_{app}=\eta_{true}$.

Capillary rheometry was carried out using a G18 needle as the capillary. The test was performed applying 5 different pressure levels for a maximum of 30 s: (i) the minimum pressure required by each sample to flow, (ii) 50 kPa (if enough to flow), (iii) 100 kPa, (iv) 150 kPa, and (v) 200 kPa (the maximum pressure provided by the printer). During this time, at least 4 samples were collected and weighed. Also, the time needed to attain the sample was measured with a stopwatch (iPhone 11, Apple, Cupertino, CA, USA). The mass flow rate was then measured as the slope of the line interpolating the extruded mass vs. time data. The volume flow rate was estimated by dividing it by the density of each suspension.

To avoid any influence of the crosslinking reaction and to obtain a better reproducibility, NA_suspensions both with a higher amount of MTZ and without it, were used, with the assumption that the liquid response before crosslinking is independent of the presence of GDL. Tests were performed at 25 °C.

#### 4.2.5. Dynamic Mechanical Analysis

Dynamic mechanical analysis was performed on unfilled and MTZ-filled hydrogels using a Modular Compact Rheometer MCR 502 (Anton Paar, Graz, Austria) in a parallel plate configuration (25 mm diameter plates, 1–2 mm gap depending on the height of hydrogel). The methods adopted were consistent with the recommendations of ASTM D4440-23 “Standard test method for plastics: dynamic mechanical properties Melts rheology” [38]. Oscillatory measurements on hydrogels were performed 24 h after GDL addition to ensure complete crosslinking. During that time, they were kept at room temperature in sealed Petri dishes.

Amplitude sweep tests were carried out in the shear amplitude range 0.01–1% at the constant frequency of 10 rad/s to determine the limit of the linear viscoelastic region (LVR), which was about 0.05%. Based on shear strain amplitude sweeps, frequency sweep measurements were performed from 1 rad/s to 100 rad/s and back to 1 rad/s for all samples at 0.01% shear strain amplitude.

The testing conditions were selected to obtain the appropriate information about structure and composition-to-mechanical property correlations, rather than to investigate the behavior of the hydrogel in mechanical conditions similar to those of the specific case of the vaginal environment.

#### 4.2.6. Samples Preparation

**Figure 13:**
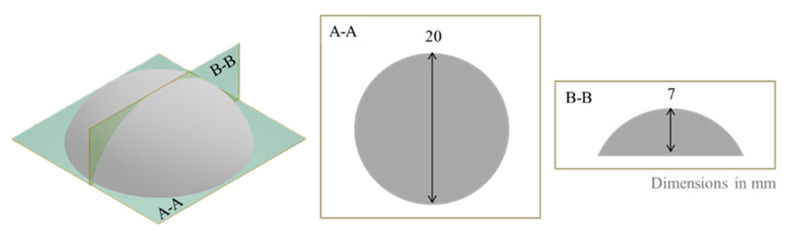
Design of dome-shaped cast sample.

The hydrogel precursors were cast in:

- custom made cylindric mold (radius = 12.5 mm, high = 1 mm) to provide samples suitable for dynamical mechanical test.
- silicon multi-portion mold with dome-shaped geometry (design shown in Figure 13) to supply samples for the swelling test. This geometry could in principle allow the estimation of both swelling and MTZ mass and volume release. The former can be obtained by mass measurement, as described below, while the latter from the optical observation of the dissolution front, exploiting the sample spherical symmetry and under the hypothesis of isotropic dissolution.

The precursor solutions were cast in the molds immediately after the addition of the GDL solution. Before using dome-shaped hydrogels for further tests, they were kept overnight at room temperature in sealed Petri dishes containing wet paper towels to keep a humid environment.

#### 4.2.7. Swelling and Stability Tests

Hydrogel samples based on all the formulations considered were cast in dedicated molds. They were submerged in 250 mL of acetate buffer at pH 4.5 (EUROPEAN PHARMACOPOEIA—4.1.3. Buffer solutions) and kept at 37 °C.

At pre-established times, the samples were removed from the buffer and weighed (E50S/3, Gibertini, Elettronica, Milano, Italy) to evaluate their stability and MTZ dissolution over time. The experiment lasted one month, during which the samples were also photographed (iPhone 11, Apple, Cupertino, CA, USA).

The mass data were used to calculate the swelling ratio *Q*, which helps quantify the medium absorption as:

(11) $$Q(t)=\frac{W_{0}-W_{\left(t\right)}}{W_{0}}$$

## Figures and Tables

**Table 1:** Cross’ model (Equation (5)) parameters of formulation containing different amounts of ALG and CaCO_3_, prepared starting from a saturated solution of MTZ.

ALG (%w/w_tot_)	CaCO_3_ (%w/w_tot_)	*η*_0_ (Pa∙s)	*m*	*λ* (s)
2	0	0.100 ± 0.003	0.4 ± 0.1	n.d.
	0.17	0.101 ± 0.003	0.4 ± 0.1	n.d.
4	0	0.86 ± 0.01	0.60 ± 0.07	n.d.
	0.34	0.88 ± 0.01	0.52 ± 0.04	0.0043 ± 0.0003
6	0	4.52 ± 0.06	0.50 ± 0.02	0.028 ± 0.002
	0.51	6.63 ± 0.05	0.470 ± 0.007	0.07 ± 0.003

**Table 2:** Cross’ model parameters of formulations containing different amounts of MTZ.

NA_	MTZ Powder, X (%V/V_tot_)	*η*_0_ (Pa∙s)	*m*	*λ* (s)
A	20	0.44 ± 0.05	1 ± 0.2	1 ± 0.5
B	20	0.88 ± 0.02	0.5 ± 0.2	n.d.
	35	10 ± 1	0.30 ± 0.03	n.d.
	50	57 ± 4	0.45 ± 0.01	0.6 ± 0.2
C	20	11.3 ± 0.4	0.48 ± 0.09	0.06 ± 0.03
	35	40 ± 2	0.53 ± 0.04	0.16 ± 0.01
	50	130 ± 9	0.56 ± 0.02	0.4 ± 0.1

**Table 3:** Herschel–Bulkley parameters for NA_A dispersion containing different amounts of MTZ powder (0–50%).

MTZ Volume Fraction, (%V/V_tot_)	*τ_y_* (Pa)	*k* (Pa∙s^n^)	*n*
0	0 ± 0.02	0.11 ± 0.01	0.93 ± 0.01
20	0 ± 0.09	0.33 ± 0.03	0.91 ± 0.02
35	0.5 ± 0.1	1.36 ± 0.06	0.81 ± 0.01
50	4.3 ± 0.4	5.7 ± 0.2	0.74 ± 0.01

**Table 4:** Thomas equation coefficients.

Formulation	*A*	*B*
NA_A	0.11 ± 0.02	11.7 ± 0.4
NA_B	0.0025 ± 0.0007	18.1 ± 0.5
NA_C	0.08 ± 0.09	9 ± 2

**Table 5:** Hydrogel formulations overview. Solutions and suspensions were prepared starting from an MTZ saturated solution.

ALG (% w/w_tot_)	CaCO_3_ (% w/w_tot_)	CaCO_3_ Suspension Code	MTZ (% V/V_tot_)	Drug-Loaded Suspension Code	Hydrogel Code
2	0.17	A	0	A.0	HA.0
			20	A.20	HA.20
			35	A.35	HA.35
			50	A.50	HA.50
4	0.34	B	0	B.0	HB.0
			20	B.20	HB.20
			35	B.35	HB.35
			50	B.50	HB.50
6	0.51	C	0	C.0	HC.0
			20	C.20	HC.20
			35	C.35	HC.35
			50	C.50	HC.50

Note: GDL was kept constant, at 1.2 w/w_tot._

## Data Availability

The data supporting the discussion and conclusions of this study are presented in the manuscript, and are available from the corresponding author upon reasonable request.

## Supplementary Materials

The following supporting information can be downloaded at: https://www.mdpi.com/article/10.3390/gels11070510/s1, Figure S1: (a) An example of viscosity curves for MTZ-saturated solution containing 4% ALG and 50% MTZ (b) the relevant average viscosity curve. Error bars represent the standard deviation.; Figure S2: Micrograph of a filled hydrogel HB.50 sample. The filler dimensions are comparable to those of the dry filler after grinding and sieving (see Figure 1a of the manuscript); Figure S3: Loss factor, tan(δ) evolution in time for HB.50. The apparent gel point can be observed for tan(δ) = 1 at about 30 minutes from non activated precursor mixing with GDL.
