# Supplementary material for: Formulation-Dependent Extrudability of Highly Filled Alginate System for Vaginal Drug Delivery
Source: Gels. 2025 Jul 1;11(7):510. doi: 10.3390/gels11070510 (PMC12295552; doi:10.3390/gels11070510)
Supplement: Supplementary file 1 [file gels-11-00510-s001.zip › gels-3707944-supplementary.pdf]

Formulation-dependent Extrudability of Highly-filled Alginate System for Vaginal Drug Delivery

Supplementary Information

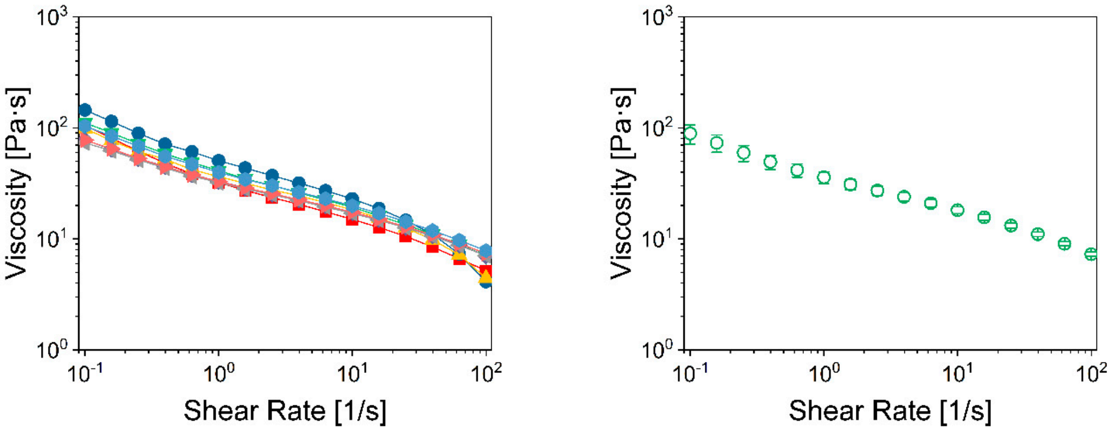

a   b

Figure S1. a) An example of viscosity curves for saturated solution containing 4% ALG and 50% MTZ b) the relevant average viscosity curves. Error bars represent the standard deviation.

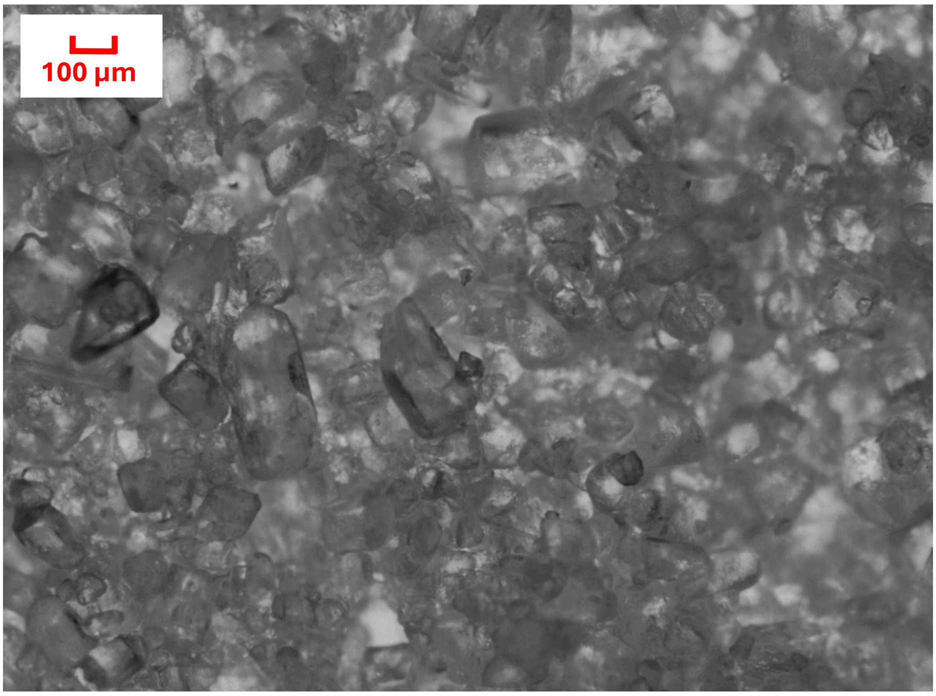

Figure S2: Micrograph of a filled hydrogel HB.50 sample. The filler dimensions are comparable to those of the dry filler after grinding and sieving (see Figure 1a)

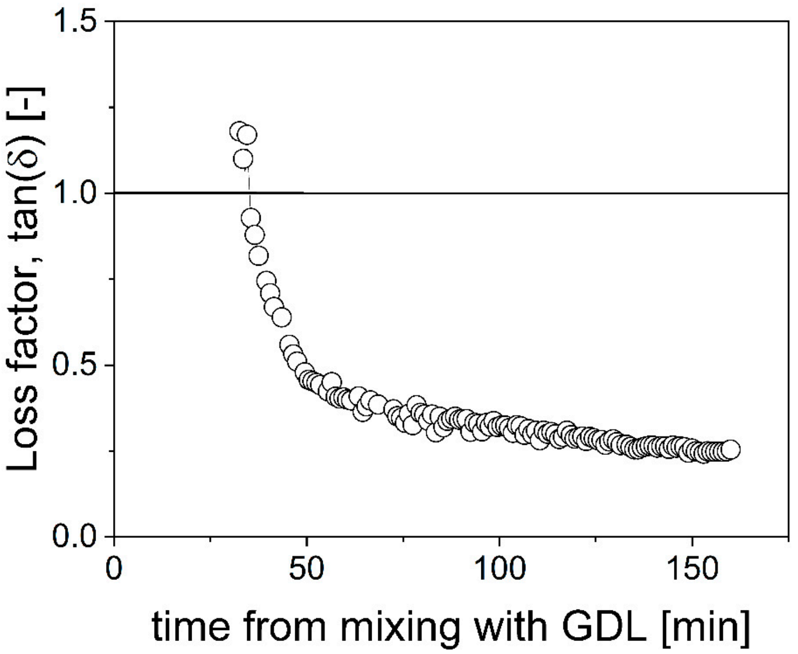

Figure S3. Loss factor, tan(δ) evolution in time for HB.50 The apparent gel point can be observed for tan(δ)=1 at about 30 minutes from non activated precursor mixing with GDL.
